# Eyespot peek‐a‐boo: Leaf rolls enhance the antipredator effect of insect eyespots

**DOI:** 10.1111/1365-2656.14232

**Published:** 2024-12-25

**Authors:** Elizabeth G. Postema

**Affiliations:** ^1^ Field Museum of Natural History Chicago Illinois USA; ^2^ Department of Entomology and Nematology, Animal Behavior Graduate Group University of California Davis California USA

**Keywords:** animal coloration, antipredator behaviour, caterpillar, deimatic display, eyespots, leaf roll, Papilionidae, spicebush swallowtail

## Abstract

1. Animal colour patterns are often accompanied by specific, synergistic behaviours to most effectively defend prey against visual predators. Given the inherent context‐dependence of colour perception, understanding how these colour‐behaviour synergies function in a species' natural environment is crucial.
2. For example, refuge‐building species create a unique visual environment where most (or all) of the body is obscured unless closely inspected. How these built environments affect the perception of defensive colour patterns by predators is not well understood.
3. Using artificial caterpillars that resemble a refuge‐building species with conspicuous markings (*Papilio troilus*; Lepidoptera: Papilionidae), I tested the hypothesis that leaf rolls amplify the antipredator effect of this species' eyespots. I compared wild avian predation rates on 659 artificial swallowtail‐like caterpillars from four treatment groups: eyespotted and non‐eyespotted, and presented in leaf rolls or on open leaves of live host plants.
4. In support of my hypothesis, eyespots only reduced predation for larvae in leaf rolls. On open leaves, eyespots had no antipredator effect. I also found that leaf rolls reduced predation in general for both eyespotted and non‐eyespotted prey.
5. These results highlight the importance of considering relevant behaviours in studies of animal coloration whenever possible, including behaviours that influence colour perception indirectly (e.g. through habitat use or modification).

## Graphical abstract

The effectiveness of antipredator colour patterns is deeply linked to prey behaviour and ‘background’ habitat; yet, little research has been done on how habitat modification shapes the perception of these signals. This study demonstrates that active environmental modification can significantly enhance the antipredator effect of eyespots in a leaf‐rolling caterpillar.

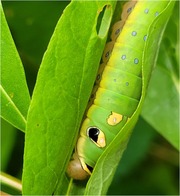

## 1 INTRODUCTION

Colour patterns in nature are diverse in form and function. For species with visual predators, body colour can help prey avoid, deter, deceive and even startle natural enemies (Cuthill et al., 2017; Huang & Caro, 2023; Postema et al., 2023). A major challenge of understanding the evolutionary forces that shape visual signals is the highly context‐dependent nature of colour. That is, the production, perception and effect of an organisms' coloration depends on more than just the light‐reflecting structures and pigments that make up physical appearance (Cuthill et al., 2017). The efficacy of antipredator coloration may be influenced by a number of other variables, such as light conditions (Endler, 1993; Nokelainen et al., 2021), habitat colour, composition and complexity (Baling et al., 2020; Geisler, 2008; Merilaita et al., 2001; Robinson et al., 2023), predator physiology and experience (Guilford & Dawkins, 1991; Mappes et al., 2014; Miller & Bee, 2012), competing ecological functions (Huang & Caro, 2023; Postema et al., 2023), and prey behaviour (Stevens & Ruxton, 2018). Prey behaviour is notable in that it can influence colour perception both directly (e.g. through the active control of colour elements; Drinkwater et al., 2022) and indirectly (e.g. by changing transmission environment, as in habitat selection or modification; Camacho et al., 2020). In recent years, there has been a push to better understand the ecology and evolution of animal coloration given these complex influences (Cuthill et al., 2017; Huang & Caro, 2023; Postema et al., 2023).

Synergies between prey behaviour and morphology can emerge at any point along the predation sequence (Kikuchi et al., 2023). Certain behaviours can help prey avoid detection altogether, such as orienting to better match substrate geometry, posing to imitate inedible objects, self‐decorating with debris, or flexibly changing colour on different backgrounds (Caro et al., 2016; Stevens & Ruxton, 2018; Suzuki & Sakurai, 2015). Many species will also deploy secondary defences once detected, approached, or touched, including fleeing, emitting sounds or chemicals, or suddenly exposing high‐contrast colours (Drinkwater et al., 2022; Kikuchi et al., 2023). The sudden exposure strategy is a type of deimatic display: that is a ‘momentary, transient, conspicuous’ signal that induces ‘a startle response’ in or overloads ‘the senses of an attacking predator’ to slow or stop the attack (Umbers & Mappes, 2016). The colour patches being revealed may or may not signal toxicity, and often involve paired eye‐like markings known as ‘eyespots’ (Drinkwater et al., 2022; Janzen et al., 2010). Examples range from the ‘blinking’ eyespot contractions of *Eumorpha* caterpillars (Hossie et al., 2013) to the flashing wings of various moth, butterfly, and mantis species (Olofsson et al., 2013; Stevens, 2005). Like other conspicuous markings (Mappes et al., 2014; Ruxton et al., 2009; Umbers et al., 2015), eyespots paradoxically risk attracting predators that are not fooled or deterred by the signal (Postema, 2021; Stevens et al., 2008). Thus, eyespotted organisms likely benefit from behaviours that limit eyespot visibility until later stages of the predation sequence (Kikuchi et al., 2023).

Janzen et al. (2010) also observe that, among lepidopterans, eyespotted larvae and chrysalises are commonly associated with dense foliage, crevices and leaf rolls. Similar to the association of eyespots with deimatic displays, these body‐obscuring habitats may help to reduce eyespot detectability until predators are directly inspecting or interacting with prey. However, our understanding of how prey behaviour, habitat characteristics and conspicuous antipredator markings (such as eyespots) interrelate is limited (Postema et al., 2023). Few experimental studies explicitly test these variables together under natural conditions, despite the fact that both transmission environment and prey behaviour are essential to how visual signals are perceived by predators (Cuthill et al., 2017; Huang & Caro, 2023; Postema et al., 2023).

**FIGURE 1:**
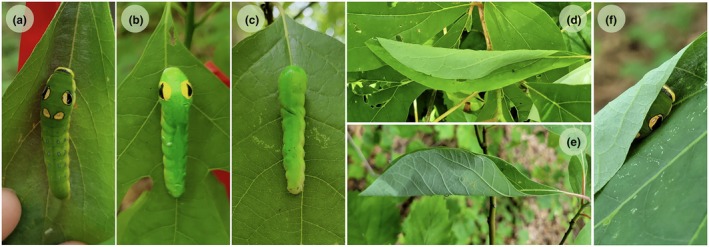
(a) A live spicebush swallowtail (*Papilio troilus*) larva on sassafras (*Sassafras albidum*). Eyespotted (b) and non‐eyespotted (c) artificial larvae on open *S. albidum* leaves. (d) A real leaf roll with a live *P. troilus* larva inside. (e) An artificial leaf roll with an artificial larva inside. (f) A live *P. troilus* larva in a leaf roll, its eyespots partially visible. Photographs by EGP.

Swallowtail caterpillars (Lepidoptera: Papilionidae) are an ideal study system to explore synergies between coloration and behaviour in the field, as they exhibit a wide variety of colour patterns (Gaitonde et al., 2018) and defensive behaviours (Hossie & Sherratt, 2013, 2014; Leslie & Berenbaum, 1990), some involving active habitat modification (Wagner, 2005). Species such as the spicebush swallowtail (*Papilio troilus*), for example, are both eyespotted (Figure 1a) and build refuges (Figure 1d). In this system, the link between behaviour and ‘background’ is explicit: the larvae use silk to construct flexible shelters from the leaves of their host plant, which they rest in during the day (Wagner, 2005). Larvae construct multiple leaf rolls over the course of their development, each taking a considerable amount of time and effort to construct (Video S1). When *P. troilus* larvae are in leaf rolls, their large, bright yellow eyespots are obscured (Figure 1d), visible only to predators that look into or open the leaf roll from the top (Figure 1f). The sudden appearance of eyespots only upon close inspection is strikingly similar to the ‘conceal‐then‐reveal’ sequence that characterizes deimatic displays (Umbers & Mappes, 2016).

I hypothesize that, for *P. troilus* larvae, environmental modification enhances the antipredator effect of their eyespots. To test this, I conducted an artificial prey experiment using a combination of eyespotted and non‐eyespotted clay caterpillars presented on either open or rolled host plant leaves (Figure 1b–e). I predicted that there would be a strong relationship between prey presentation (roll vs. open leaf) and colour (eyespots vs. no eyespots). Specifically, I expected eyespots to reduce predator attacks on caterpillars in leaf rolls to a greater extent than eyespots on exposed caterpillars. Based on previous research on other leaf‐rolling arthropods, I also predicted an overall protective effect of leaf rolls (Murakami, 1999; Tvardikova & Novotny, 2012). To my knowledge, this is the first study to test how environmental modification impacts the efficacy of a conspicuous antipredator visual signal, under natural field conditions.

## 2 MATERIALS AND METHODS

### 2.1 Site description and host plant selection

I conducted field predation trials at two sites in Ann Arbor, MI, approximately 4 km apart (Bird Hills Nature Area, “Bird Hills”: 42°18′09.1″ N 83°45′37.9″ W; Nichols Arboretum, “Arboretum”: 42°16′48.9″ N 83°43′20.5″ W; Figure S1). This study did not require ethical approval, and was conducted with written permission from both the University of Michigan and the Ann Arbor Parks department (no specific permits were needed). Both sites were mixed coniferous‐deciduous forests, and contained host plants of *P. troilus* caterpillars such as sassafras (*Sassafras albidum*) and tulip tree (*Liriodendron tulipifera*). I selected a total of 421 individual plants for prey deployment (189 *S. albidum* and 232 *L. tulipifera*) across the two sites, with at least 5 m between each plant. Plants had to be small enough to be searchable by hand (less than ~300 cm) and sturdy enough to support artificial prey (at least 10 cm, with at least one fully developed leaf). I checked all plants for *P. troilus* larvae before the start of each trial and omitted occupied plants (*n* = 17; Figure S1). Potential avian predators of *P. troilus* were present at the sites throughout the experiment (Table S1), as well as live *P. troilus* larvae (Table S2, Figure S1) and adults. I ran two predation trials at each site, from 3–12 July 2021 and 20–30 July 2021. In 2022, I surveyed natural leaf rolls at both sites to characterize their arthropod communities (Figure S4).

### 2.2 Host plant and habitat measurements

To account for the influence of the transmission environment, I measured plant height and canopy openness for each host plant I used during the experiment. For plant height, I took measurements (in cm) from ground‐level at the base of the stem to the tip of the highest leaf. I measured canopy openness by taking skyward photos with a 180° hemispheric lens directly above each plant. I then processed these photos using ImageJ (version 1.53) to calculate the proportion of open sky relative to vegetative cover in each image. I used canopy cover as a proxy for lighting conditions, as this gives a more holistic view of week‐to‐week light levels compared to a single light measurement taken for each plant.

### 2.3 Artificial prey construction and deployment

I constructed artificial *P. troilus* larvae by pressing white modelling clay (Van Aken Plastalina^TM^) into 3D‐printed moulds. The resulting models were 4 cm long, approximately the size of a fourth or fifth instar larva (Figure 1a). To attach prey to host plants, I inserted a loop of 26‐gauge craft wire into each clay caterpillar, leaving the two ends of the loop exposed on the ventral side. I applied three layers of acrylic airbrush paint (CREATEX tan, yellow‐green and dark green) to create the appearance of green countershading, which is an important aspect of visual defence for many swallowtail species that can interact with eyespots (Rowland et al., 2007). Using yellow and black acrylic paint, I hand‐painted eyespots on half of the prey (“eyespotted”, Figure 1b), while leaving the rest blank (Figure 1c). I sprayed each model with one coat of Krylon^®^ matte finish spray to prevent excessive shine and protect the paint from weather damage.

I randomly assigned one of the four treatments (Figure 1) to each plant, ensuring that there were equal proportions of each prey type for both host plant species. For the open leaf treatment group, I attached artificial prey to host plant leaves by poking the loose wire‐ends of each prey through the leaf, then twisting them tightly around the midrib. For the prey in leaf rolls, I folded the leaf over the artificial prey and secured it shut with a strip of Scotch^TM^ double‐sided tape (Figure 1e). I positioned all prey with the “head” pointed up towards the leaf petiole, which reflects this species' typical resting position (Figure 1a,f; Table S2). I selected the artificial prey's location on the plant haphazardly and measured prey height (in cm) from the ground to the top of the caterpillar's head. Before the start of the trial, I took a photo of each artificial caterpillar in place.

### 2.4 Artificial prey collection and scoring

I collected artificial prey after approximately 5 days of exposure (mean: 121 h, SD: 8 h). At the end of each trial, I inspected prey for evidence of predation. Avian and mammalian attacks are clearly distinguishable by the shape of the bite‐marks in the clay (Figure S2). I recorded any missing prey (that could not be found after carefully scouring a 1 m^2^ area around the original location) as attacked by an unknown predator. I photographed all recoverable prey with visible attack marks. I excluded a few cases (*n* = 9) where either the artificial caterpillar or the entire leaf roll fell from the plant with no sign of predator damage. I also excluded one artificial caterpillar in which a real *P. troilus* caterpillar had crawled into the artificial leaf roll, and three artificial prey where the host plant could not be located. In total I deployed 809 artificial caterpillars. Of those, I included data from 659 artificial caterpillars in the main analysis, excluding prey attacked by non‐avian predators. While mammals are important insect predators, they represented only a small percentage of attacks in this study (~5%). Conceptually, I was most interested in the predation patterns of highly visual predators like birds, which also tend to hunt diurnally, when *P. troilus* are resting in leaf shelters (Hossie & Sherratt, 2012, 2013; Nyffeler et al., 2018). Additionally, mammalian and unknown predators showed little variation in predation between treatments (Figure S3). As it is difficult to detect evidence of arthropod attacks using clay caterpillars, I did not consider these types of predators in my analysis.

### 2.5 Visual modelling

I measured the reflectances of real and artificial *P. troilus* caterpillars (body and eyespots), as well as fresh *S. albidum* leaves, using an Ocean Optics Flame Miniature (FLAME‐S‐UV‐VIS‐ES) spectrometer with Ocean Optics PX‐2 Pulsed Xenon light source, calibrated with a 99% Labsphere reflectance standard (Figure 3). To model how live and artificial prey on host plant leaves might look to an avian predator, I used the package pavo in R to analyse the resulting reflectance data (Maia et al., 2019). While I did not directly observe predator attacks in this study, chickadees, tits and other birds in the family Paridae are common predators of lepidopteran larvae (Naef‐Daenzer & Keller, 1999). Black‐capped chickadees (*Poecile atricapillus*) were abundant at both study sites across the experimental trials (Table S1), so I chose to model the vision of a species in the same family, the Eurasian blue tit (*Cyanistes caeruleus*). To calculate photoreceptor quantum catches from my reflectance spectra, I used the pavo function ‘vismodel’ and arguments based on a similar example case (Maia et al., 2019): visual = “uv”, achromatic = “bt.dc”, relative = FALSE.

To compare live and artificial prey coloration as seen on a typical background (host plant leaves) by an avian predator, I calculated pairwise colour distances using the pavo function ‘coldist’, again set to arguments based on the example case: achromatic = TRUE, *n* = c(1, 2, 2, 4), weber = 0.1, weber.achro = 0.1. I then averaged the colour distance (ΔS) for each comparison and compared the resulting values to a standard colour discriminability threshold of Δ*S* = 1. This threshold represents when two colours become just distinct enough to tell apart by the modelled viewer, also known as a ‘just‐noticeable‐difference’ (JND; Kelber et al., 2003; Vorobyev et al., 2001). I did not test for statistical differences in the mean ΔS values of artificial versus live prey, as differences in values greater than one are difficult to interpret without further behavioural data from the relevant receiver(s) (Santiago et al., 2020).

### 2.6 Statistical analysis

To analyse these data, I used binomial generalized linear models with a complementary log–log link function in R (ver. 1.1.463). For all models, I set avian predation as the binomial response variable (0 = not attacked, 1 = attacked) and included days exposed as an offset term. For the first model, I included the following independent variables: trial, location, leaf roll treatment (rolled, open), colour treatment (eyespotted, non‐eyespotted), canopy openness (a proportion, from 0 = sky fully obscured by canopy to 1 = open sky), plant height and prey height (in cm). I also tested for an interaction between the roll treatment and colour treatment. To determine the statistical significance of each independent variable across the model, I used likelihood ratio tests (package lmtest). As leaf roll treatment and the colour*roll interaction term were both statistically significant, I constructed additional models without the interaction term to test for the simple effects of colour and leaf rolls, also using likelihood ratio tests.

## 3 RESULTS

### 3.1 Effect of colour and leaf rolls on predation

The overall avian predation rate on artificial caterpillars was 13.4% over a 5‐day period. Predation did not vary significantly by trial (χ^2^ = 0.95, df = 1, *p* = 0.33) or location (χ^2^ = 0.96, df = 1, *p* = 0.33). There was a significant interaction between leaf rolls and eyespots (χ^2^ = 4.64, df = 1, *p* < 0.05): in leaf rolls, eyespots reduced the probability of predation (7.1% reduction, χ^2^ = 6.31, df = 1, *p* < 0.05). On exposed prey, eyespots had no significant effect on predation (χ^2^ < 0.01, df = 1, *p* = 0.98). Overall, leaf rolls significantly reduced predation relative to prey on open leaves (12.9% reduction, χ^2^ = 23.09, df = 1, *p* < 0.001). This was true of both eyespotted (17.3% reduction, χ^2^ = 21.93, df = 1, *p* < 0.001) and non‐eyespotted prey (8.4% reduction, χ^2^ = 5.36, df = 1, *p* < 0.05).

### 3.2 Effect of habitat structure on predation

In general, canopy cover was dense (mean: 14.3% open sky, SD: 8%). Plants used in the experiment were an average of 124.9 cm tall (SD: 60.1 cm), with a maximum height of 315 cm and minimum height of 31 cm. Prey were placed 11.0–281.0 cm high on plants (mean: 83.5, SD: 44.1 cm), comparable to the heights of live *P. troilus* caterpillars I observed in the field (25–164 cm, mean: 83.8, SD: 40.5 cm; Table S2). Ultimately, none of these variables had a significant effect on artificial caterpillar predation (canopy openness: χ^2^ = 0.12, df = 1, *p* = 0.73; plant size: χ^2^ = 0.11, df = 1, *p* = 0.74; prey height: χ^2^ = 1.30, df = 1, *p* = 0.52).

### 3.3 Colour analysis of real and artificial caterpillars

Average colour distances (Δ*S*) from sassafras leaves differed between the bodies of live (mean Δ*S*: 3.6, SD: 0.4) and artificial caterpillars (mean Δ*S*: 5.5, SD: 0.4). This was also true of live (mean Δ*S*: 6.9, SD: 0.5) and model eyespots (mean Δ*S*: 8.6, SD: 0.4) compared to sassafras leaves. However, all mean Δ*S* values were greater than 1 (Figure 3), meaning avian predators would find the bodies and eyespots of both live and artificial prey visually distinct from the leaves of a common *P. troilus* host plant (Vorobyev et al., 2001; Vorobyev & Osorio, 1998).

## 4 DISCUSSION

**FIGURE 2:**
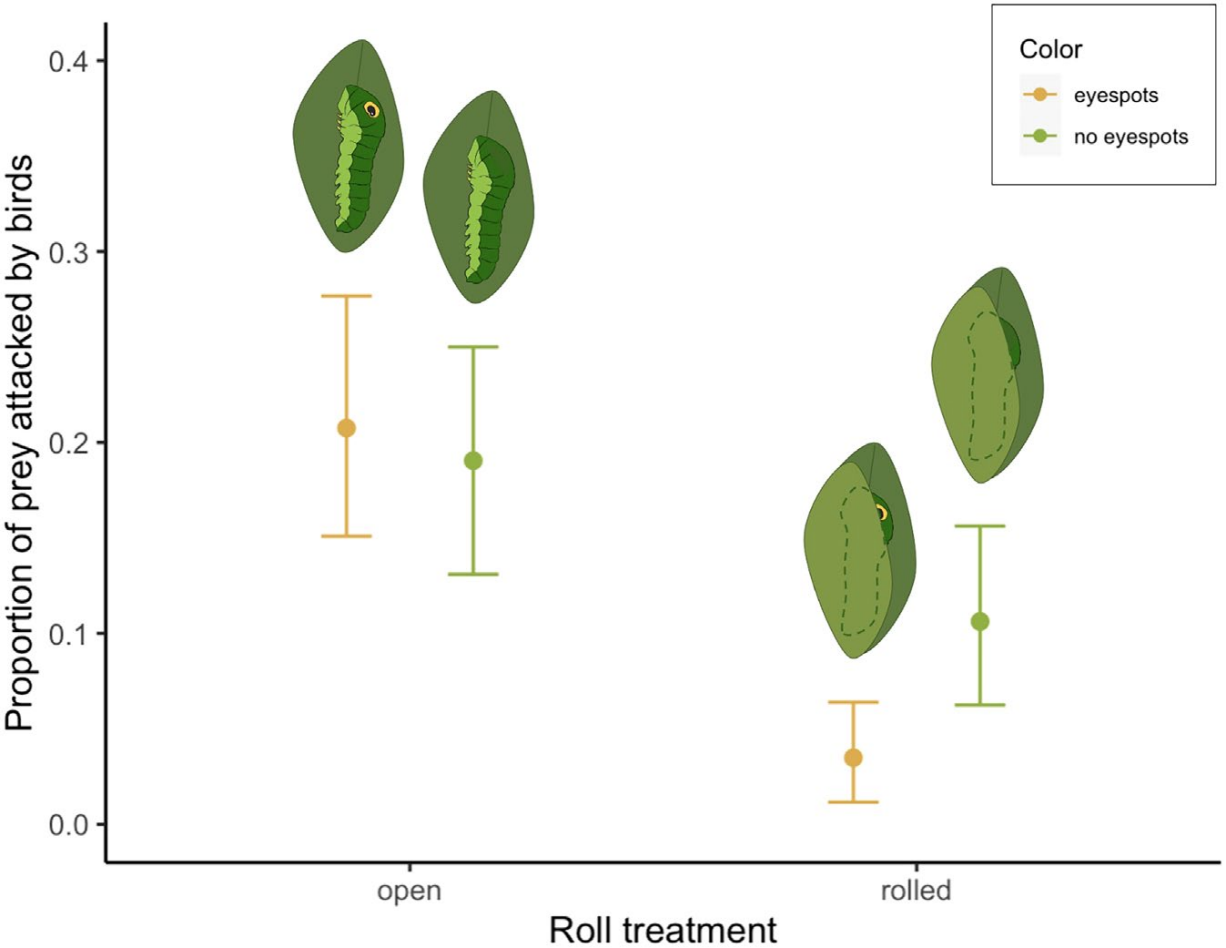
Mean proportions of artificial prey in each treatment group attacked by avian predators, ±SE (*n* = 659). Yellow points represent eyespotted prey, while green points represent non‐eyespotted prey. Illustrations by Mia Lippey.

For *P. troilus* caterpillars, leaf rolls are key to the antipredator function of eyespots (Figure 2). Specifically, in this study, eyespots *only* reduced avian predation when artificial prey were presented in leaf rolls. This reduction in predation was sizable: from 20.8% attacked on open leaves to only 3.5% attacked in leaf rolls, comparing among prey with eyespots. This result supports the hypothesis that environmental modification (such as refuge‐building) can shape the nature and effectiveness of conspicuous visual signals. In other arthropods, leaf rolls have been shown to provide protection against various natural enemies (LoPresti & Morse, 2013; Murakami, 1999; Tvardikova & Novotny, 2012), but the present study is the first to demonstrate that a built environment can reduce predation *through* its effect on a visual signal.

While the specific combination of leaf‐rolling and eyespots may be uncommon (but see *Cephise nuspesez* and *Ridens panche*, family Hesperiidae: Lepidoptera; Janzen et al., 2010), habitat modification, in general, is not. Habitat modification and structure‐building is taxonomically widespread, and organisms engage in these behaviours for various reasons (Hansell, 2007). By contrast, research on how these structures might affect an organism's visual appearance is limited (but see Hultgren & Stachowicz, 2011). Perhaps the strongest evidence for animals actively shaping visual signals via habitat modification comes from studies on elaborate avian mating displays. For example, male gold‐collared manakins (*Manacus vitellinus*) create ‘courts’ free from litter to improve the visual impact of their brightly coloured plumage (Uy & Endler, 2004), while male bowerbirds (family Ptilonorhynchidae) construct forced‐perspective illusions to increase the attractiveness of their bowers' decor (Kelley & Endler, 2017). The current study adds to this small but growing body of experimental work that explicitly links animal morphology, behaviour and transmission environment in the context of visual signalling.

A plausible mechanism for the colour‐behaviour synergy observed in this study is that leaf rolls create a ‘conceal‐then‐reveal’ sequence, much like a deimatic display (Umbers & Mappes, 2016). Eyespots that become apparent *only* when predators are at close range have two potential benefits: (1) preventing unnecessary detection (a risk for any conspicuous colour pattern; Ruxton et al., 2009) and (2) improving the mimetic and/or startling effect of the eyespots themselves (Hossie & Sherratt, 2014; Janzen et al., 2010). Alternatively, these results could be explained by a kind of perspective‐dependent aposematism or Batesian mimicry (Barnett et al., 2017; McEwen et al., 2024). Predators may not be startled per se, but simply wary of high‐contrast patterning on an organism whose body (and thus identity) is mostly concealed. Of course, these mechanisms are not necessarily mutually exclusive. Predators with different levels of experience may perceive *P. troilus* eyespots as aposematic, mimetic, deimatic or some combination (De Bano et al., 2015; Mappes et al., 2014; Stevens, 2005; Stevens & Ruxton, 2014). Further field experiments, with direct observations of predators, would be useful to clarify how eyespots in refugia are perceived.

Regardless of mechanism, there is a clear advantage of concealing and/or deimatic behaviours for eyespotted organisms (Drinkwater et al., 2022; Kikuchi et al., 2023). Without leaf rolls, the large, conspicuous eyespots of artificial caterpillars in this study had no antipredator effect (Figure 2). Interestingly, while eyespots are widespread among late‐instar swallowtail caterpillars, leaf‐rolling is not (Wagner, 2005). As leaf rolls stand out from other foliage (Kobayashi et al., 2020) and frequently contain edible arthropods (Vieira & Romero, 2013; Figure S4), their eyespot‐enhancing effect may be counterbalanced by their attractiveness as foraging microhabitats in some species (Kikuchi et al., 2023). However, many eyespotted swallowtail larvae do create simple silk mats to rest on and also tend to inhabit dense, broad‐leaved host plants (Gaitonde et al., 2018; Wagner, 2005). Refuge‐building, then, likely represents one of many ways to reduce the risk of long‐range conspicuousness among eyespotted organisms, from associating with body‐obscuring microhabitats (Janzen et al., 2010) to covering up colour patches when not in active use (Drinkwater et al., 2022).

**FIGURE 3:**
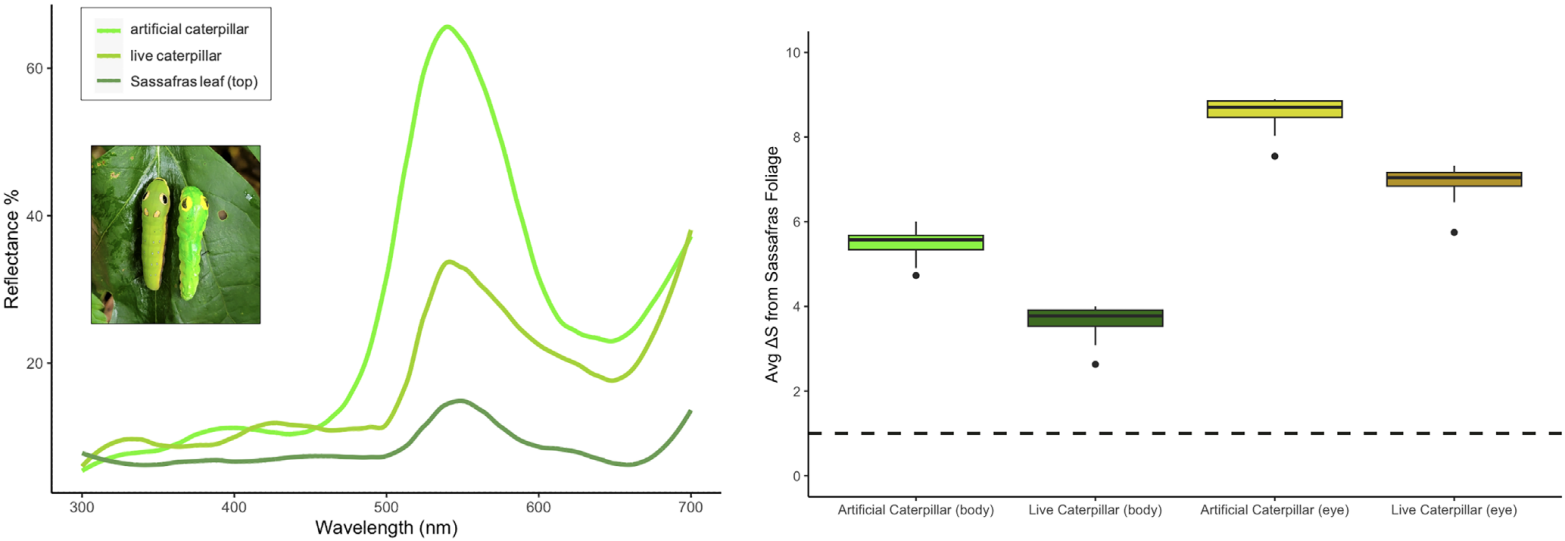
(Left) Reflectance spectra measured from the tops of sassafras leaves (*n* = 10) and the green areas of artificial caterpillars (*n* = 3) and live caterpillars (*n* = 3). (Right) Mean colour distances (Δ*S*) of four colour types (the bodies and eyespots of live and artificial caterpillars) compared to sassafras leaves, ±SE. Colour distances are modelled using the visual physiology of the Eurasian blue tit, *Cyanistes caeruleus*. The dashed line marks the just‐noticeable‐difference (JND) threshold at Δ*S* = 1. Values above the line represent two colours that can be reliably discriminated by the receiver.

Alternatively, it is possible that the eyespots of *artificial* caterpillars are simply less effective than the eyespots of live caterpillars when openly displayed. This could stem from problems with model coloration (e.g. unnaturally saturated paint leading to greater detectability; Stoddard et al., 2018), or the fake caterpillars' inability to actively respond to predators (Paluh et al., 2014). Via spectral analysis, I found that avian predators would be able to easily distinguish both live and fake caterpillars from host plant foliage (Kelber et al., 2003; Vorobyev et al., 2001; Vorobyev & Osorio, 1998). Compared to host plant leaves, both caterpillar types were above the JND threshold of Δ*S* = 1 (Figure 3). As ΔS does not necessarily scale linearly with conspicuousness beyond this threshold (Cheney et al., 2019; Green et al., 2022; Santiago et al., 2020), it is not clear how much more detectable fake prey were compared to real prey, if at all. In terms of the inanimate nature of artificial prey (Paluh et al., 2014), it is possible that including the full suite of swallowtail defensive behaviours would have increased eyespot effectiveness on exposed surfaces (Hossie & Sherratt, 2013; Leslie & Berenbaum, 1990). Typical swallowtail defensive responses include thorax inflation, gentle swaying, and/or extending the forked osmeterium (scent gland; Table S3). However, while eyespots and thorax inflation can both reduce avian predation on swallowtail‐like prey, these effects appear to be independent rather than synergistic (Hossie & Sherratt, 2013). Further, there is no evidence that osmeterium eversion deters avian predators, as this is more likely a chemically based defence against arthropods (Leslie & Berenbaum, 1990). While I do not discount the simple effects these behaviours may have on predation, or the utility of multicomponent defences (Kikuchi et al., 2023; Skelhorn et al., 2016), leaf rolls likely enhance eyespots regardless of additional defences.

The results of this field experiment demonstrate that prey can shape the effectiveness of visual signals through habitat modification. The influence of prey behaviour on colour defences may not always be as obvious as actively flashing colour patches, self‐decorating (Ruxton & Stevens, 2015) or changing colour (Duarte et al., 2017); in some cases, prey behaviour may operate on signal perception indirectly. At a broader level, this research highlights the need for a holistic approach when studying the ecology and evolution of visual signals. Ultimately, it is essential to consider colour patterns less as static traits, and more as multivariate optima: that is complex strategies that may involve selection on morphology, behaviour or structures beyond the body of the organism (Huang & Caro, 2023; Laland, 2004; Postema et al., 2023; Stuart‐Fox, 2022).

## CONFLICT OF INTEREST STATEMENT

The author reports no conflicts of interest.

## Supporting information


**Table S1.** Bird species surveys conducted at Bird Hills Nature Area (BH) and Nichol's Arboretum (Arb) directly after each Trail (post‐trial 1: 7/14, post‐trial 2: 7/30).
**Table S2.** Observations and measurements of live *P. troilus* larvae and their host plants.
**Table S3.** Behavioral observations of live *P. troilus* larvae in response to disturbance (i.e. when I opened their leaf roll).
**Figure S1.** A map of the locations used in the field predation trials.
**Figure S2.** Locations of observed predator (avian, mammalian, and unknown) attacks on artificial prey at Bird Hills (left panels) and Nichols Arboretum (center panels), for trial 1 (top panels) and trial 2 (bottom panels).
**Figure S3.** Proportion of rolled and unrolled artificial prey attacked by mammal (left) and unknown (right) predators, ±SE (*n* = 725).
**Figure S4.** (Left) Total counts of each organism type found in surveys of naturally occurring leaf rolls (*n* = 464). (Right) Examples of naturally occurring leaf rolls observed in the field.

**Video S1.** Sped‐up timelapse of a late‐instar spicebush swallowtail caterpillar (*P. troilus*) constructing a new leaf roll on sassafras (*Sassafras albidum*). The entire video was recorded over the course of 70 min. Videography by EGP.

## Data Availability

Data available from the Dryad Digital Repository: https://doi.org/10.5061/dryad.3j9kd51vz (Postema, 2024).
