# Supplementary material for: Eyespot peek‐a‐boo: Leaf rolls enhance the antipredator effect of insect eyespots
Source: J Anim Ecol. 2024 Dec 25;94(5):814–22. doi: 10.1111/1365-2656.14232 (PMC12056355; doi:10.1111/1365-2656.14232)
Supplement: Supplementary file 1 — Table S1. Bird species surveys conducted at Bird Hills Nature Area (BH) and Nichol's Arboretum (Arb) directly after each Trail (post‐trial 1: 7/14, post‐trial 2: 7/30). Table S2. Observations and measurements of live P. troilus larvae and their host plants. Table S3. Behavioral observations of live P. troilus larvae in response to disturbance (i.e. when I opened their leaf roll). Figure S1. A map of the locations used in the field predation trials. Figure S2. Locations of observed predator (avian, mammalian, and unknown) attacks on artificial prey at Bird Hills (left panels) and Nichols Arboretum (center panels), for trial 1 (top panels) and trial 2 (bottom panels). Figure S3. Proportion of rolled and unrolled artificial prey attacked by mammal (left) and unknown (right) predators, ±SE (n = 725). Figure S4. (Left) Total counts of each organism type found in surveys of naturally occurring leaf rolls (n = 464). (Right) Examples of naturally occurring leaf rolls observed in the field. [file JANE-94-814-s001.pdf]

Table S1. Bird species surveys conducted at Bird Hills Nature Area (BH) and Nichol's Arboretum (Arb) directly after each Trail (post-trial 1: 7/14, post-trial 2: 7/30). The author conducted 30-minute visual and aural surveys for 5 total subsites (200m^2 rectangles; 3 in BH, 2 in Arb). Counts for each species are recorded below, but are a rough estimate, as it was not always possible to tell if individuals had been repeatedly observed. *Plausible predators of *P. troilus*, based on size and diet information listed on allaboutbirds.org.

Common Name	Scientific Name	BH 7/14	Arb 7/14	BH 7/30	Arb 7/30
*American robin	*Turdus migratorius*	11	11	7	8
*Black-capped chickadee	*Poecile atricapillus*	15	2	6	4
*Blue jay	*Cyanocitta cristata*	7	4	10	3
*Cedar waxwing	*Bombycilla cedrorum*	0	0	0	1
*Eastern towhee	*Pipilo erythrophthalmus*	0	2	0	0
*Eastern wood-peewee	*Contopus virens*	8	2	4	2
*European starling	*Sturnus vulgaris*	2	0	0	0
*Gray catbird	*Dumetella carolinensis*	0	1	0	0
*Northern cardinal	*Ailuroedus jobiensis*	3	1	9	4
*Scarlet tanager	*Piranga olivacea*	2	0	0	0
*Song sparrow	*Melospiza melodia*	0	1	0	0
*Tufted titmouse	*Baeolophus bicolor*	1	0	0	0
*Yellow warbler	*Setophaga petechia*	0	1	0	0
American goldfinch	*Spinus tristis*	2	3	9	6
Chimney swift	*Chaetura pelagica*	0	1	0	0
Downy woodpecker	*Dryobates pubescens*	4	0	3	1
Field sparrow	*Spizella pusilla*	0	1	0	0
House finch	*Haemorhous mexicanus*	1	0	1	0
Northern flicker	*Colaptes auratus*	3	0	4	0
Pileated woodpecker	*Dryocopus pileatus*	1	1	0	0
White-breasted nuthatch	*Sitta carolinensis*	3	2	0	2
Tree swallow	*Tachycineta bicolor*	0	0	1	1
Red-bellied woodpecker	*Melanerpes carolinus*	0	0	0	1
Red-tailed hawk	*Buteo jamaicensis*	0	0	4	0

Table S2. Observations and measurements of live *P. troilus* larvae and their host plants. In total, I observed n = 17 live larvae throughout the experiment. I observed some larvae multiple times; date observed is recorded in parentheses within the column (m/d). *Height is measured from the ground to the position of the larva in its host plant. **Larval orientation within the leaf roll: up = towards leaf petiole, down = away from leaf petiole, towards the ground.

ID	Host Plant	*Height (cm)	Larva Length (cm)	**Orientation
1	spicebush	121	2.5 (6/17), 3.5 (6/24)	up (6/17)
2	sassafras	-	-	up
3	sassafras	164	3.4 (6/24)	up (6/24)
4	sassafras	54	2.45 (6/26), 3.3 (6/28), 4.3 (7/2)	up (6/26), up (6/28), up (7/2)
5	sassafras	136	0.7 (6/28)	up (6/28)
6	sassafras	103	0.5 (6/28), 1.7 (7/7)	up (6/28), up (7/7)
7	sassafras	70	3.0 (6/28)	up (6/28)
8	sassafras	35	1.7 (6/28), 2.0 (7/2)	up (6/28)
9	sassafras	71	2.4 (6/28)	up (6/28)
10	sassafras	34	3.0 (6/28), 3.5 (7/2)	up (6/28), up (7/2)
11	sassafras	35	1.0 (6/28), 2.1 (7/2), 2.2 (7/7)	down (6/28), up (7/2), up (7/7)
12	sassafras	69	0.8 (6/28), 1.9 (7/7)	up (6/28), up (7/77)
13	sassafras	96	0.8 (6/28)	up (6/28)
14	sassafras	25	4.0 (7/8)	up (7/8)
15	sassafras	119	2.0 (7/11), 2.8 (7/19), 1.2 (7/26)	up (7/11)
16	sassafras	99	1.2 (7/11)	up (7/11)
17	sassafras	91	2.1 (7/12)	up (7/12)

Table S3. Behavioral observations of live *P. troilus* larvae in response to disturbance (i.e., when I opened their leaf roll). In total, I observed n = 17 live larvae throughout the experiment; some were observed multiple times. M = ambulatory movement, O = osmeterium extended, P = thorax puffed up, S = swaying back and forth, NR = no response. Dash = no recorded observation.

ID	First Observation	Second Observation	Third Observation
1	O, P	P, S	P
2	NR	-	-
3	M, P	-	-
4	slight P, slight S	NR	-
5	P, S	-	-
6	P, S	slight P	-
7	slight P	-	-
8	M, P	slight S	-
9	M, S	-	-
10	some M, P	P	-
11	O, P	NR	P
12	NR	O, P	-
13	P	-	-
14	slight P	-	-
15	M, O, P	P, S	-
16	slight P	-	-
17	slight P	-	-

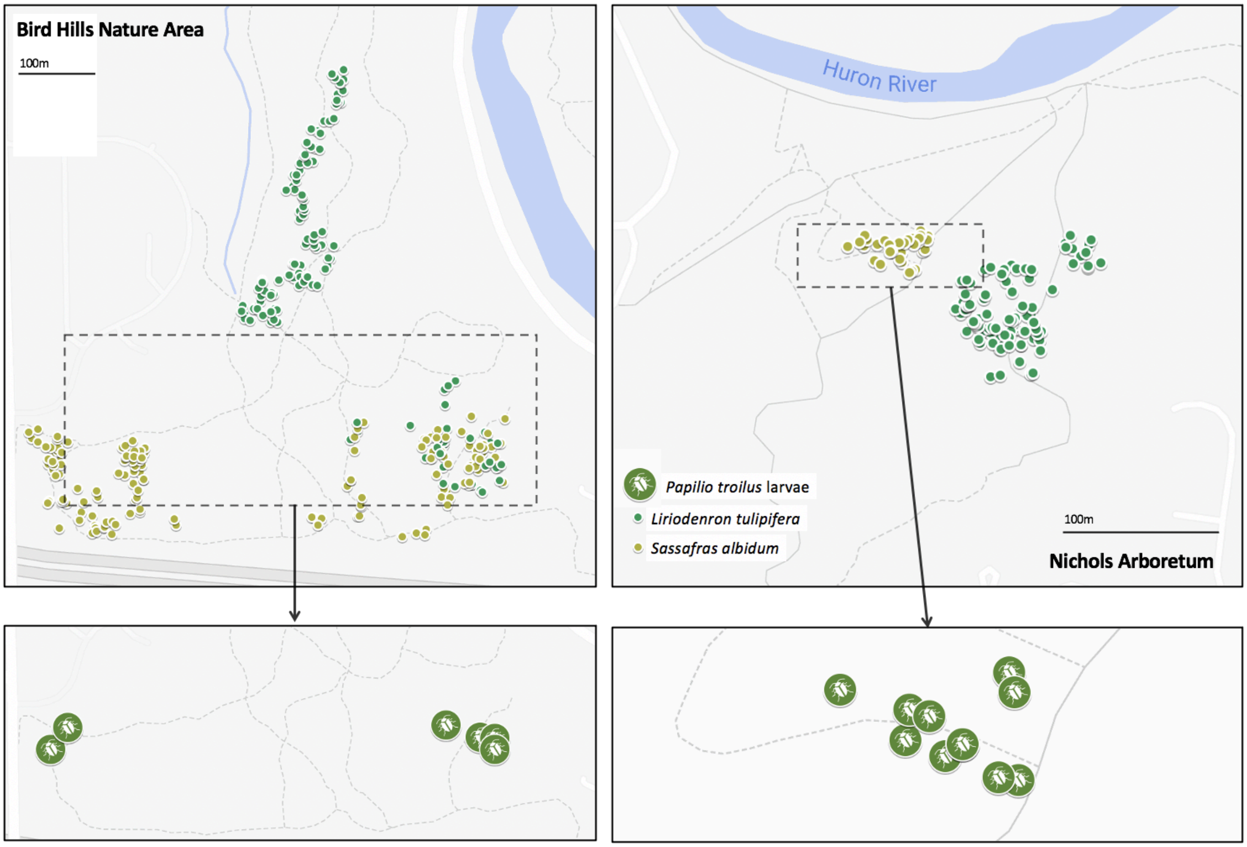

Figure S1. A map of the locations used in the field predation trials. On the left, individual plants used at Bird Hills Nature Area; on the right, plants used at Nichol's Arboretum. Individual host plants are marked with yellow-green (*L. tulipifera*) or dark green (*S. albidum*) circles. Below each map are close-up sections with circular icons. Each of these represents the locations of a live *P. troilus* caterpillar observed just before and during the experiment.

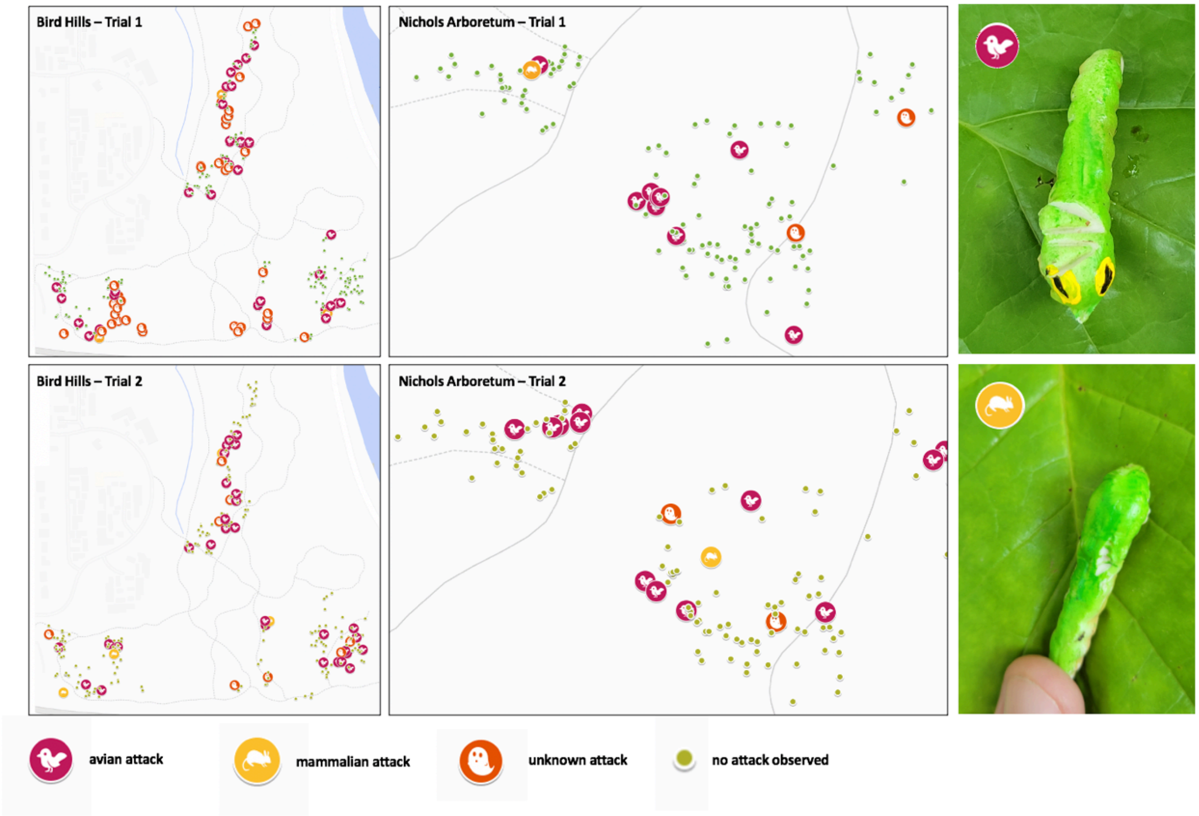

Figure S2. Locations of observed predator (avian, mammalian, and unknown) attacks on artificial prey at Bird Hills (left panels) and Nichols Arboretum (center panels), for trial 1 (top panels) and trial 2 (bottom panels). Panels on the right show examples of avian (top) versus mammalian (bottom) predation marks. Photographs by EGP.

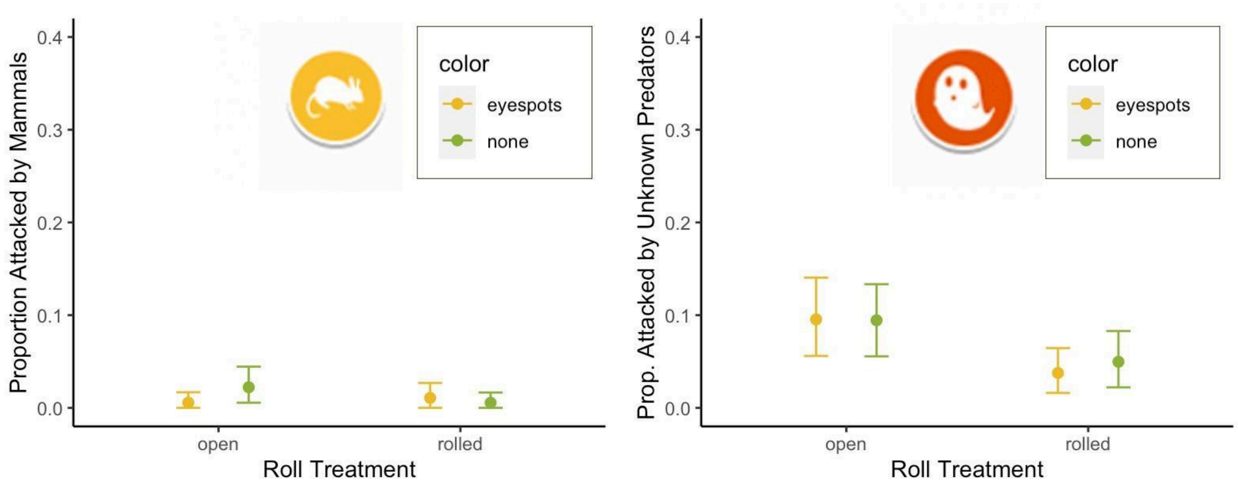

Figure S3. Proportion of rolled and unrolled artificial prey attacked by mammal (left) and unknown (right) predators, ± SE (n = 725). Yellow points represent eyespotted prey, while green points represent non-eyespotted prey.

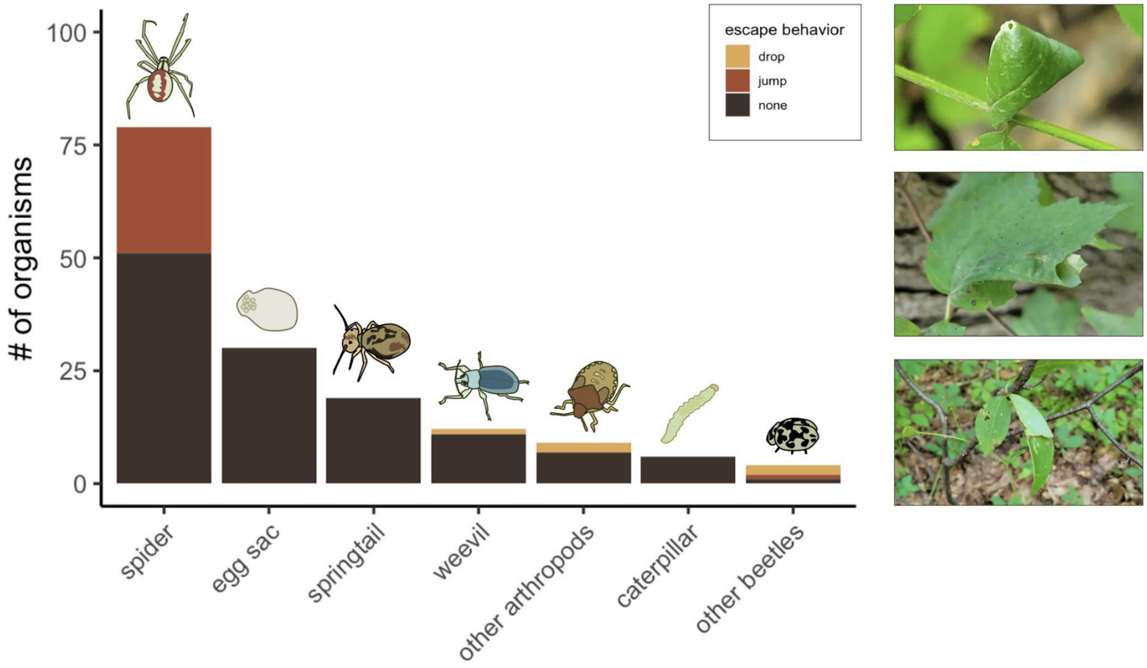

Figure S4. (Left) Total counts of each organism type found in surveys of naturally occurring leaf rolls (n = 464). Within each organism category, counts of individuals that displayed escape behaviors in response to the leaf roll being disturbed (dropping, jumping, or no response) are represented in yellow, red, and brown, respectively. (Right) Examples of naturally occurring leaf rolls I observed in the field; leaf rolls varied in size, structure, and plant species. Photographs and illustrations by EGP.

__**Survey Method:**__ I conducted surveys of naturally occurring leaf rolls at Bird Hills from August 23rd-27th, 2022. Surveys were conducted along five 50m linear transects that overlapped with areas previously used for the predation experiment. I surveyed all plants of a specific height range (over 0.5m and under 2m tall) within one meter of the transect for rolled or folded leaf structures. This height range reasonably overlaps with the height range of natural swallowtail leaf rolls I found (25-164cm), and thus where I expected avian predators might forage for prey in

leaf rolls. Plants were marked as having either 0, 1, 2, or >3 leaf rolls. Leaf rolls varied in structure but were counted in the survey as long as they were (a) at least partially enclosed, and (b) clearly constructed by an animal. I recorded the contents of up to 3 leaf rolls per plant by carefully opening each structure by hand. Organisms were visually identified to broad taxonomic groups (e.g., spiders, springtails, beetles). Rolls with either non-living debris or nothing inside were recorded as "empty." As some organisms immediately jumped or dropped out of the rolls, I also recorded the flight behavior of all observed organisms: jumping, dropping, or no response. In total, I surveyed 464 leaf rolls across 457 individual plants. Of these plants, ~10% were also host plants of *P. troilus,* including spicebush (*Lindera benzoin),* sassafras, and tulip tree.

Video S1: Sped-up timelapse of a late-instar spicebush swallowtail caterpillar (*P. troilus*) constructing a new leaf roll on sassafras (*Sassafras albidum*). The entire video was recorded over the course of 70 minutes. Videography by EGP.
